# Mapping and Characterization of Local Structures of CsPbBr_3_

**DOI:** 10.1021/acsomega.4c04354

**Published:** 2024-08-09

**Authors:** Tahira Khan, Sviatoslav Baranets, Manas R. Gartia, Jianwei Wang, Jyotsna Sharma

**Affiliations:** † Department of Petroleum Engineering, Louisiana State University, Baton Rouge, Louisiana 70803, United States; ‡ Department of Chemistry, Louisiana State University, Baton Rouge, Louisiana 70803, United States; § Department of Mechanical and Industrial Engineering, Louisiana State University, Baton Rouge, Louisiana 70803, United States; ∥ Department of Geology and Geophysics, Louisiana State University, Baton Rouge, Louisiana 70803, United States; ⊥ Center for Computation and Technology, Louisiana State University, Baton Rouge, Louisiana 70803, United States

## Abstract

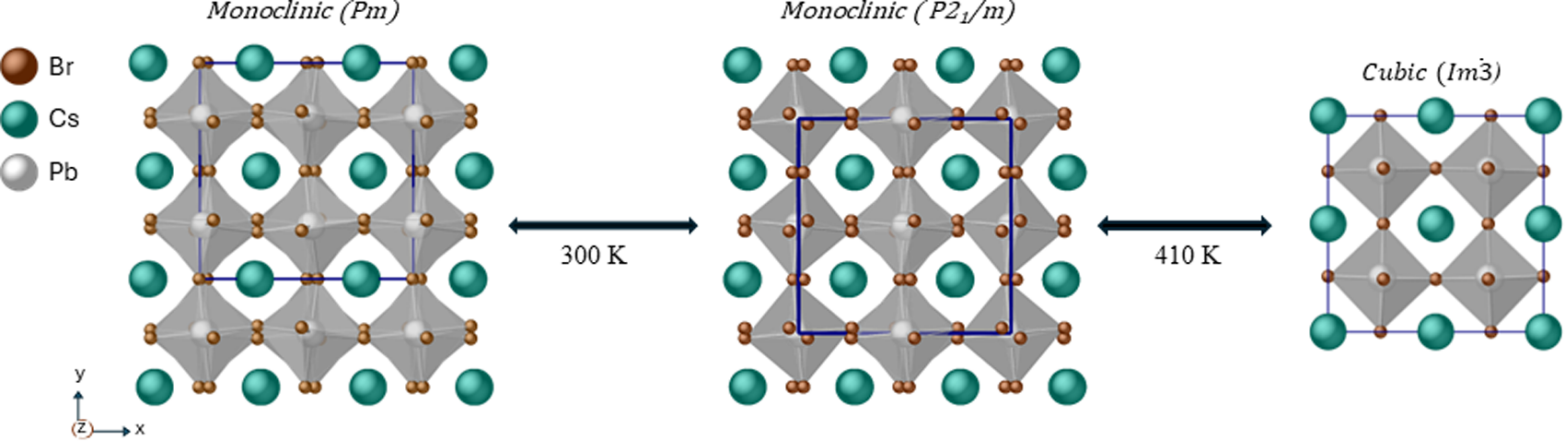

Inorganic perovskite CsPbBr_3_ is a promising material for optoelectronic applications and high-energy radiation detection due to its excellent photophysical properties, high carrier mobility, large carrier diffusion length, and higher stability than organic perovskite materials. Understanding phase transitions at the atomic level is crucial for optimizing its applications. Here, we employ experimental characterizations and molecular dynamics simulations to study the phase transitions in CsPbBr_3_ as a function of temperature. The simulation results are compared with the experimental results, which include X-ray diffraction (XRD). Our simulations provide new insights into the electronic structure and dynamic behavior of the Cs, Pb, and Br atoms as a function of temperature. We observe distinct phase transitions from monoclinic to cubic and analyze the associated changes in the local environment through atomic density contour maps. Our analysis of the atomic density distributions of the Pb, Br, and Cs atoms provides information about the crystal symmetry as a function of temperature. The tilt and rotation angles of [PbBr_6_] octahedra are increasing with the temperature increase and are found nonzero above 410 K when the structure is cubic, exhibiting the presence of dynamic tilting. Overall, our findings shed light on the thermal stability and structural dynamics of CsPbBr_3_, contribute to the fundamental understanding of its phase behavior, and provide a crucial pivot for guiding the design of next-generation optoelectronic and radiation detection devices.

## Introduction

Organic and inorganic halide-based perovskite materials (represented as ABX_3_, where A^+^ can be an organic or inorganic cation, B^2+^ is a transition or main group metal cation, and X = Cl^–^, Br^–^, or I^–^) have been widely studied for their remarkable applications in solar cells and high-energy-radiation detection due to their excellent photophysical properties and low-cost fabrication process.^1−15^ Among these perovskite materials, the inorganic lead halide-based material, cesium lead bromide (CsPbBr_3_), has been widely studied because of its capability toward γ-ray detection^6,13,16^ due to its high carrier mobility, large carrier diffusion lengths,^17,18^ and higher stability than organic perovskite materials at high temperatures.^5,7,9^ The ideal structure of perovskite is cubic, which is the stable structure CsPbBr_3_ adapts at high temperatures.^19,20^ At low temperatures, CsPbBr_3_ undergoes a few phase transitions. The temperature-dependent phase transition in CsPbBr_3_ has been widely studied, and different phases have been reported based on both experimental and computational approaches.^19−23^ The materials’ physical properties depend on the crystal structure. So, understanding the crystal structure and phase transition of the material is crucial for understanding structure–property relationships and, therefore, for developing devices for specific applications under various environmental conditions.

The initial experimental work based on the X-ray diffraction analysis revealed that CsPbBr_3_ adopts a cubic crystal system above 410 K and is monoclinic at room temperature (RT).^24^ Later, studies showed that CsPbBr_3_ crystallizes in the orthorhombic space group *Pnma* at RT and undergoes the phase change to tetragonal (*P*4/*mbm*) at around 361 K and to cubic (*Pm*3̅*m*) at around 410 K.^19−23,25−28^ Most of these studies suggest that the stable phase of CsPbBr_3_ is orthorhombic from RT down to 4 K, while some reports show that it undergoes several phase transitions at low temperatures.^19,24,25,27,29,30^ A recent study based on high-resolution synchrotron single-crystal X-ray diffraction (SCXRD) revealed that the high-temperature stable phase of CsPbBr_3_ is cubic of the space group (*Im*3̅) above 410 K; it undergoes a structural transition to monoclinic (the space group *P*2_1_/*m*) between ≈410 and ≈300 K and another transition to monoclinic (the polar group *Pm*) below ≈300 K, respectively.^31^ In addition to these experimental studies, much work has been performed on this material based on density functional theory (DFT). However, the focus is on the structural, electronic, optical, mechanical, and thermoelectric properties. There is a lack of understanding of the octahedral orientation and tilt dynamics and their correlation with the phase transitions as a function of temperature.

CsPbBr_3_ is sensitive to external factors like stress in addition to temperature.^9,32−39^ Such external factors may impact the local structures such as the displacement of Cs cations or the rotation of the octahedral cage occupied by Pb cations and Br anions. These external factors may also affect the electronic structures such as the band gap edge associated with Br and Pb orbitals. These interactions may give rise to the octahedra’s rotation and tilt, and their dynamics can affect photon absorption. Therefore, it is crucial to understand the material’s local atomistic and electronic structure under such factors for its specific applications in devices.

In this work, CsPbBr_3_ samples were synthesized using a solution-based method and characterized by experiments that included powder X-ray diffraction (PXRD), SCXRD, and thermogravimetric analyses (TGA). Ab initio molecular dynamics (AIMD) simulations are highly suitable for understanding the materials’ electronic and molecular structures. Results from simulations at temperatures from 50 to 530 K provide essential information about the dynamics of the phase changes by mapping the atomic density of individual atoms and calculating several factors like lattice parameters and the tilt and rotation angles of the octahedra, in addition to the lattice dimensions. The atomic density contour maps, the lattice parameters, tilt, and rotation angles of the octahedra as a function of temperature offer a clear view of the structure changes and connect these changes with the phase transitions.

## Methods

### Sample Preparation

**Figure 1:**
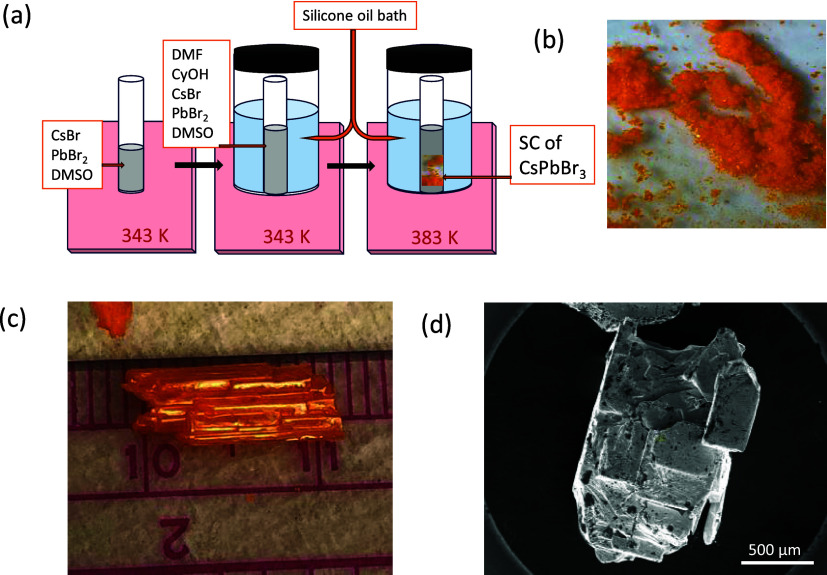
(a) Schematic of the synthesis of CsPbBr_3_ by the ITC method. (b, c) Optical images of the single crystals of CsPbBr_3_ (“Photograph courtesy of “T. Khan”. Copyright 2024.”) and (d) SEM image of a CsPbBr_3_ single crystal (“Photograph courtesy of “Benjamin Maerz”. Copyright 2024.”).

The synthesis of CsPbBr_3_ single crystals is performed by the inverse temperature crystallization (ITC) process reported elsewhere.^16^ A precursor solution containing cesium bromide (CsBr) and lead bromide (PbBr_2_) in a 1:2 ratio was prepared in dimethyl sulfoxide (DMSO) at 343 K in a vial and filtered. The solution was kept at 343 K, and a mixture of 5.1 g of cyclohexanol (CyOH) and 9.1 g of dimethylformamide (DMF) was added drop by drop to the solution, while the precursor solution was constantly stirred. After that, the mixture was placed in a silicone oil bath at 343 K to maintain a uniform temperature across the solution in the vial, and it was heated to 383 K at the rate of 3 K/h. During the heating process, many small crystals were formed. The temperature of the solution was maintained at 383 K for 12 h. Unfortunately, this process did not yield any crystals of large size. The precursor solution with small single crystals of CsPbBr_3_ was cooled to 323 at 20 K/h. The orange crystals were collected, washed with DMF, and dried in air. Figure 1a summarizes the procedure for the crystals’ growth. Figure 1b shows small single crystals of CsPbBr_3_. We utilized a Thermo Scientific (TFS Helios G5 PFIB CXe) scanning electron microscope equipped with an OXFORD Instruments Ultim Max Detector spectrometer to analyze the sample. An operational acceleration voltage of 2 kV was used to collect the scanning electron microscopy (SEM) image, which is shown in Figure 1d. Figure 1c depicts the single crystal obtained at 383 K using reduced amounts of DMF and CyOH in the precursor solution. The crystal exhibits an elongated length along one axis compared to the second axis and is shortest along the third axis.

### X-ray Diffraction (XRD)

**Figure 2:**
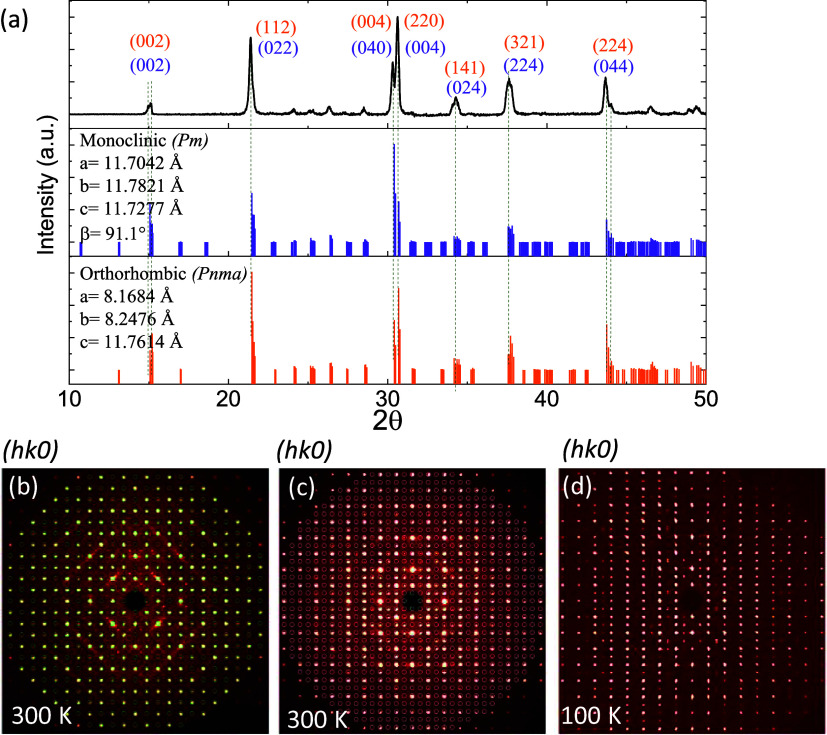
(a) Powder XRD pattern collected at RT. The bottom panels show XRD peaks obtained from simulations for the CsPbBr_3_ orthorhombic phase (the space group *Pnma*) and the monoclinic phase (*Pm*). The peaks are indexed with (*hkl*) values for the orthorhombic and monoclinic phases. Synthesized precession images of a crystal of CsPbBr_3_ at 300 K fitted for (b) orthorhombic and (c) monoclinic crystal systems, respectively. Weak diffraction spots with half-integer values are observed for the precession image generated for the orthorhombic cell. Unfitted reflections can be indexed on a primitive monoclinic supercell (Table S1). (d) Precession image generated for the 100 K dataset indicates a phase transition to the monoclinic crystal system accompanied by modulation.

Powder X-ray diffraction (PXRD) measurements were carried out on a Rigaku Miniflex 600 diffractometer, with filtered Cu Kα radiation of wavelength λ = 1.5418 Å, at room temperature. Single crystals of CsPbBr_3_ were ground in an agate mortar and pestle to take the PXRD pattern. Data were collected between 5 and 75° in 2θ with a step size of 0.02° at a speed of 3 s per step counting time. The PXRD pattern was matched with those generated theoretically using VESTA for orthorhombic and monoclinic structures reported in refs (27,31), respectively, as shown in Figure 2a.

We conducted structural analysis utilizing a single-crystal X-ray diffraction (SCXRD) technique. Data were collected with 50 K increments from 100(2) to 300(2) K using a Bruker D8 Venture DUO with a Photon III C14 detector diffractometer equipped with Ag Kα radiation (λ = 0.56086 Å). The additional data set was collected at 280(2) K. A properly sized individual crystal of CsPbBr_3_ was chosen, trimmed to the required dimensions while being submerged in Paratone-N oil, and positioned on a low-background MiTeGen plastic loop connected to the goniometer head. Temperature regulation was achieved through a continuous flow of cold nitrogen gas. The procedures for collecting intensity data, reducing data, refining cell parameters, and correcting for absorption were all managed using the APEX5 software suite.^40,41^ The crystal structures were solved employing the intrinsic phasing technique via SHELXT and subsequently refined utilizing full-matrix least-squares methods on *F*^2^ with SHELXL. The graphical interface Olex2 facilitated these procedures.^42−44^Tables S1 and S3 present selected details of data collection and pertinent crystallographic parameters.

### Thermogravimetric Analysis (TGA)

Thermogravimetric analysis (TGA) was conducted by using a TA Instruments Discovery TGA550 instrument with a nitrogen purge flow rate of 100 mL/min. The sample underwent heating to 873 K at a rate of 10 K/min. The decomposition temperature (*T*_d_) was determined by identifying the onset point of the maximum weight loss rate.

### Differential Scanning Calorimetry (DSC)

Differential scanning calorimetry (DSC) experiments were performed using a TA Instruments Discovery DSC250 instrument under a nitrogen flow rate of 50 mL/min, with Tzero aluminum pans employed. The experimental procedure comprised the following steps: (1) equilibration at 233 K followed by ramping to 423 K at a rate of 10 K/min; (2) ramping from 283 K down to 233 K; (3) further ramping to 673 K at 10 K/min; (4) subsequent ramping down to 233 K at 10 K/min; and (5) final ramping to 233 K at 10 K/min.

### Ab Initio Molecular Dynamics Simulations (AIMD Simulations)

Density functional theory-based first-principles molecular dynamics (MD) simulations were performed using the generalized gradient approximation (GGA)^45^ and the projector-augmented wave method^46^ as implemented in VASP.^47^ A plane-wave basis set with an energy cutoff of 400 eV, a γ point Brillouin zone sampling, and a Perdew–Burke–Ernzerhof (PBE) exchange–correlation functional were used. The valence state has one electron with a core electron configuration of 5s^2^5p^6^6s^1^ for Cs, two electrons with a core 6s^2^6p^2^5d^10^ for Pb, and seven electrons with a core 4s^2^4p^5^ for Br. The CsPbBr_3_ crystal system was initially relaxed at 0 K and 0 GPa. Electronic structure calculations, including density of states (DOS) and charge densities, were conducted with the relaxed structure. This relaxed cell dimension was used to model the effect of temperature on the crystal structure from 50 to 520 K. The MD runs were performed with a time step of 3 fs and run durations varied from 5 to 20 ps depending on temperature, with longer durations corresponding to the lower temperatures. Initial configurations were discarded, and averages were calculated for the equilibrium simulations.

### Analysis of the Tilt and Rotation Angles

The schematic for the tilt and rotation angle calculations is shown in the insets of Figure 6a,b. The tilt angle is calculated as the angle formed by Br–Br–Br atoms, as shown in the inset of Figure 6a.^48^

In a perovskite structure, when the octahedra are nonrotated, the Br atom lies on the center of the line connecting two Pb atoms positioned on opposite sides relative to that Br atom. This means that the Pb–Pb–Br angle is 0° in a nonrotated structure. When the octahedra are rotated, the Br atom no longer lies on the line connecting the Pb atoms positioned on its opposite sides. This changes the Pb–Pb–Br bond angle from 0°. The rotation of the octahedra can therefore be analyzed by measuring the deviation of the Pb–Pb–Br angle from 0°.^49^

### Atomic Density Contour Maps

The XDATCAR file generated during the MD simulations in VASP carries information about the ionic configuration of the system as a function of time. The atomic density contour maps were obtained from the data stored in the XDATCAR file using the Python code developed for this study.

## Results and Discussion

### Structural Analysis of CsPbBr_3_ Single Crystals

The structural analysis of CsPbBr_3_ is performed by PXRD and SCXRD. Figure 2 shows the PXRD pattern collected at room temperature and SCXRD precession images generated for data collected at 300 and 100 K. As shown in Figure 2a, the XRD patterns simulated for orthorhombic (*Pnma*) and monoclinic (the space group *Pm*) structures generated by VESTA provided in refs (27,31) are also shown. The PXRD peaks are indexed with Miller indices (*hkl*) for both simulated structures. The experimental pattern resembles both simulated XRD patterns for the orthorhombic substructure with unit cell parameters *a* ≈ √2*a*_p_, *b* ≈ √2*a*_p_, and *c* ≈ 2*a*_p_ and monoclinic *a* ≈ 2*a*_p_, *b* ≈ 2*a*_p_, and *c* ≈ 2*a*_p_, β ≠ 90° (where *a*_p_ is the basic unit cell parameter for a cubic perovskite structure, *a*_p_ ≈ 5.8 Å) with the glazer notation *a*^–^*b*^–^*c*^–^. Both patterns are practically indistinguishable due to the negligible observed intensity of low-angle superstructure peaks, complicating the unambiguous determination of the crystal system.

To identify the presence of superstructure peaks and gain insights into the presence of monoclinic structures, we collected SCXRD data at various temperatures. Upon analysis of the data collected at 300 K, we could identify two types of unit cells, with *a*, *b*, and *c* parameters of ca. √2*a*_p_, √2*a*_p_, and 2*a*_p_ and ca. 2*a*_p_, 2*a*_p_, and 2*a*_p_, respectively, corresponding to the orthorhombic substructure and monoclinic superstructure. The former smaller unit cell is determined if only high-intensive reflections are selected for the unit cell determination, whereas the latter larger cell is identified if weak reflections are considered as well (shown in Figure S1). These observations corroborate well with the results reported in ref (31), where the monoclinic superstructure was suggested for CsPbBr_3_ below 300 K. Figure 2b,c show the precession images generated along (*hk*0*l*) at 300 K for orthorhombic and monoclinic models. These reciprocal lattice images for the orthorhombic model indicate the presence of weak half-integer reflections (Figure 2b) that can, however, be described by the monoclinic supercell (Figure 2c). The monoclinic distortion is well pronounced at 300 K, evidenced by the deviation of the β angle from 90° (Tables S1 and S2).

We can readily solve the crystal structure using both models, orthorhombic *Pnma* and monoclinic *Pm*. However, the superstructure reflections indicate that the latter is preferred, as it aligns with earlier reports.^31^ Both structures are practically identical, with a significant distortion of the [PbBr_6_] octahedra, which corroborates well with our calculations that we will discuss later under the section “Phase Transition and Local Structure of CsPbBr_3_”. Upon further cooling down of the crystal, the monoclinic unit cell becomes more apparent, as shown in Figure 2d,e and Table S2. The low-temperature phase transition is accompanied by severe modulation, which is well pronounced for the 100 K data set.^31^

**Figure 3:**
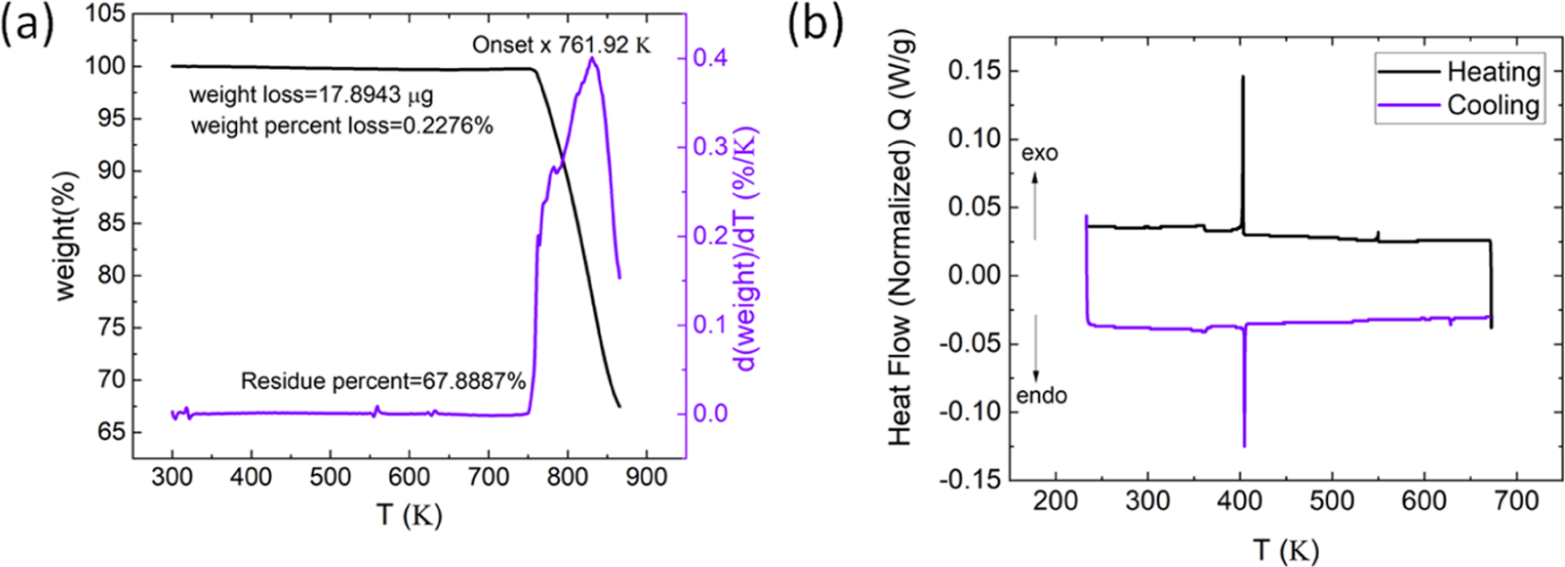
Thermal analysis of CsPbBr_3_ conducted using the ITC method, illustrating (a) TGA analysis and (b) DSC analysis.

The TGA and DSC analyses of the CsPbBr_3_ crystals are shown in Figure 3a and b, respectively. CsPbBr_3_ prepared by the ITC method is stable up to 761.92 K, while 33% of mass loss was seen at the melting temperature (840 K).^7,14^ DSC analysis shows a prominent change in phase at 410 K, which is the transition temperature to cubic, consistent with other reports.^7,14,15^ However, a small peak starting at 351 K was also observed. According to ref (31), CsPbBr_3_ undergoes a first-order phase transformation at 410 K from cubic (*Im*3̅) to monoclinic (*P*2_1_/*m*), showing a first-order isostructural transition found at 350 K that could be compared to the peak appearing at 351 K in DSC analysis.^31^

### Electronic Structures of CsPbBr_3_

**Figure 4:**
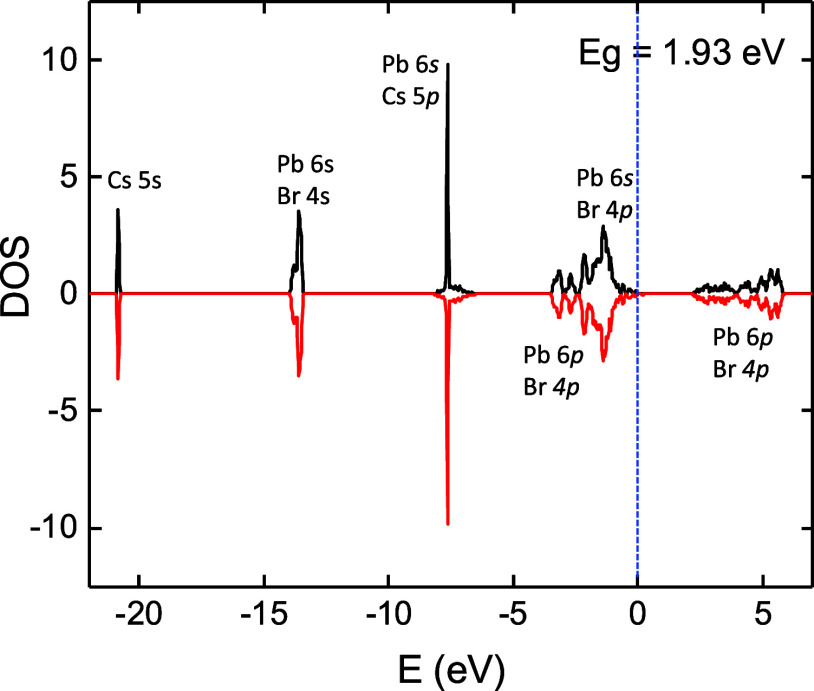
Total DOS for CsPbBr_3_ depicting a band gap energy of 1.93 eV. The black curves represent spin-up states, while the red curves represent spin-down states. A vertical dashed line indicates the Fermi level.

Figure 4 presents the total density of states (DOS) of CsPbBr_3_, computed by using density functional theory (DFT), starting from a tetragonal structure configuration. Energy minimization was performed. The calculated band gap energy is 1.93 eV, comparable to the other reports.^50−52^ The band at −7.5 eV arises from Cs 5p–Pb 6s orbitals. Such an overlap between Cs and Pb has been reported previously in refs (50−52). The top of the valence band shows Pb 6s–Br 4p and Pb 6p–Br 4p overlapping. The bottom of the conduction band (2–6 eV) arises from the overlapping Pb 6p–Br 4p orbitals.

**Figure 5:**
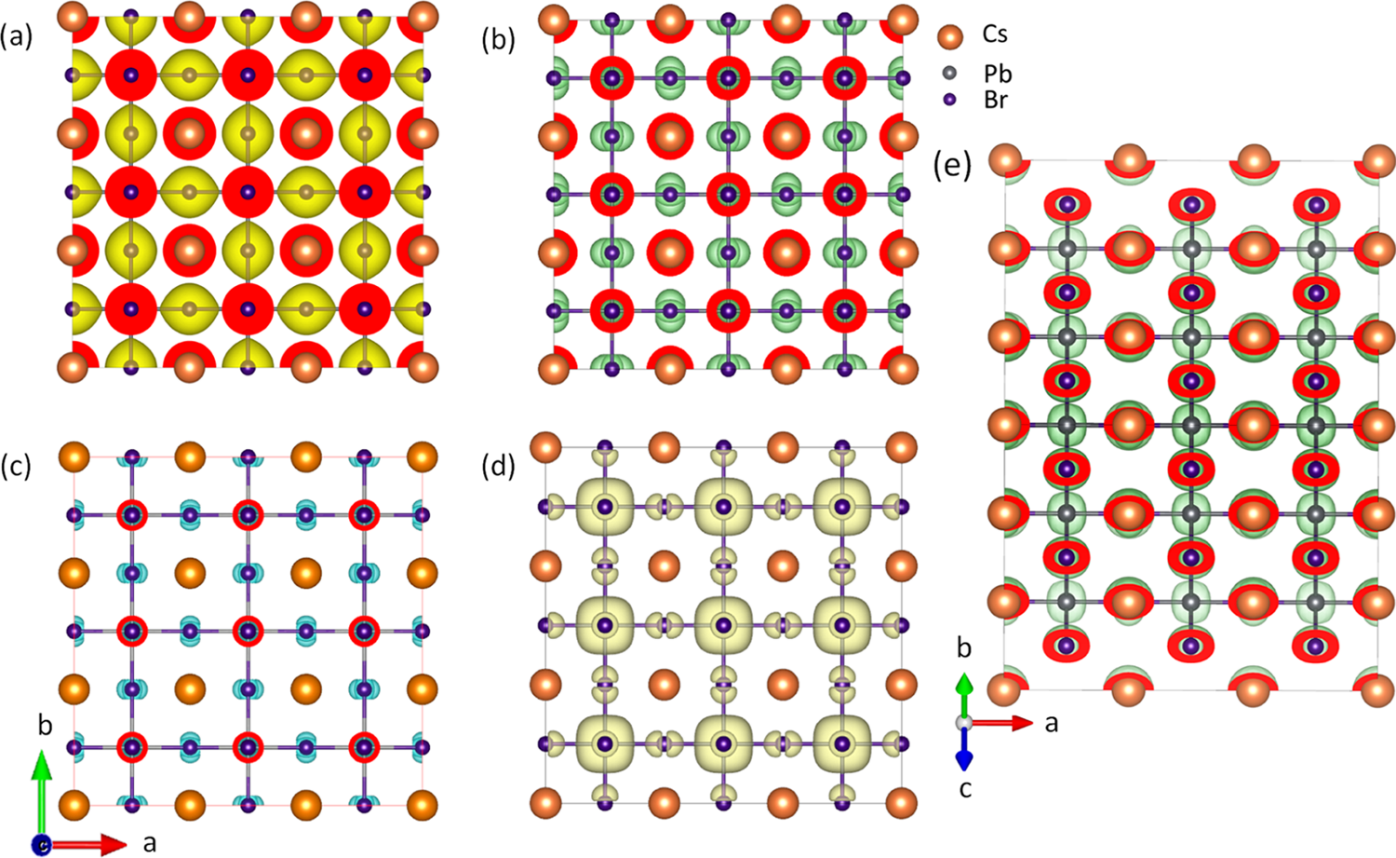
(a) Calculated charge density (an isosurface value of 0.03 e/Å^3^) and (b) electron localization function (ELF) (an isosurface value of 0.85) for CsPbBr_3_. In panel (a), the electron density is highlighted in yellow, with red indicating cross-cuts at the atoms. (b) Electron localization function (in green), with a side view (panel (e)), emphasizing the Pb atom and its ELF. (c, d) Charge density at the top of the valence band (from −3.5 eV to the Fermi level) and at the bottom of the conduction band (from 2 to 7 eV), respectively, with isosurface values of 0.10 and 0.01 e/Å^3^. Note the absence of visible Pb atoms except in panel (e).

Figure 5a illustrates the total charge density as an isosurface plot. The charge density of Cs is isotropic, reflecting the nature of its s orbit. For Br, the isosurface is elongated along the coordinate axis, reflecting the nature of the p orbit. For Pb, the isosurface is elongated along the neighboring Br atoms (shown in the Supporting Information Figure S3), reflecting the nature of its p orbit.

The electron localization function (ELF) serves to measure the concentration of the electron pair localization within a system. It evaluates the probability of locating an electron near a specified point while considering its spin. Essentially, the ELF evaluates the degree of electron localization within a specific spatial region. This approach facilitates the characterization of electron pair distribution within complex, multielectronic systems. As the functions are formulated, an ELF value close to one corresponds to a region in the atomic space where electrons are highly localized (paired), whereas a value close to half corresponds to a gaslike behavior. As seen in Figures 5b and S3b, the elongated Br ELF with the Pb atoms indicates a strong electron localization and a significant presence of the covalent bond between Br and Pb atoms.

The partial charge density isosurface is plotted for the energy range from −3.5 eV to the Fermi level (shown in Figures 5c and S3c) and from 2 to 7 eV (Figure 5d). Figure 5c shows the electron density isosurface of electrons at the top of the valence bands. Figures 5d and S3d show the electron density isosurface of electrons at the bottom of the conducting bands. The electron charge densities for the top of the valence band (spherical for 6s-Pb and dumbbell for 4p-Br) and the bottom of the conducting band (anisotropic for 6p-Pb and dumbbell for 4p-Br) (Figure 5c) are consistent with the nature of their orbital hybridization.

### Phase Transition and Local Structure of CsPbBr_3_

#### Crystal Lattice Changes as a Function of Temperature

**Figure 6:**
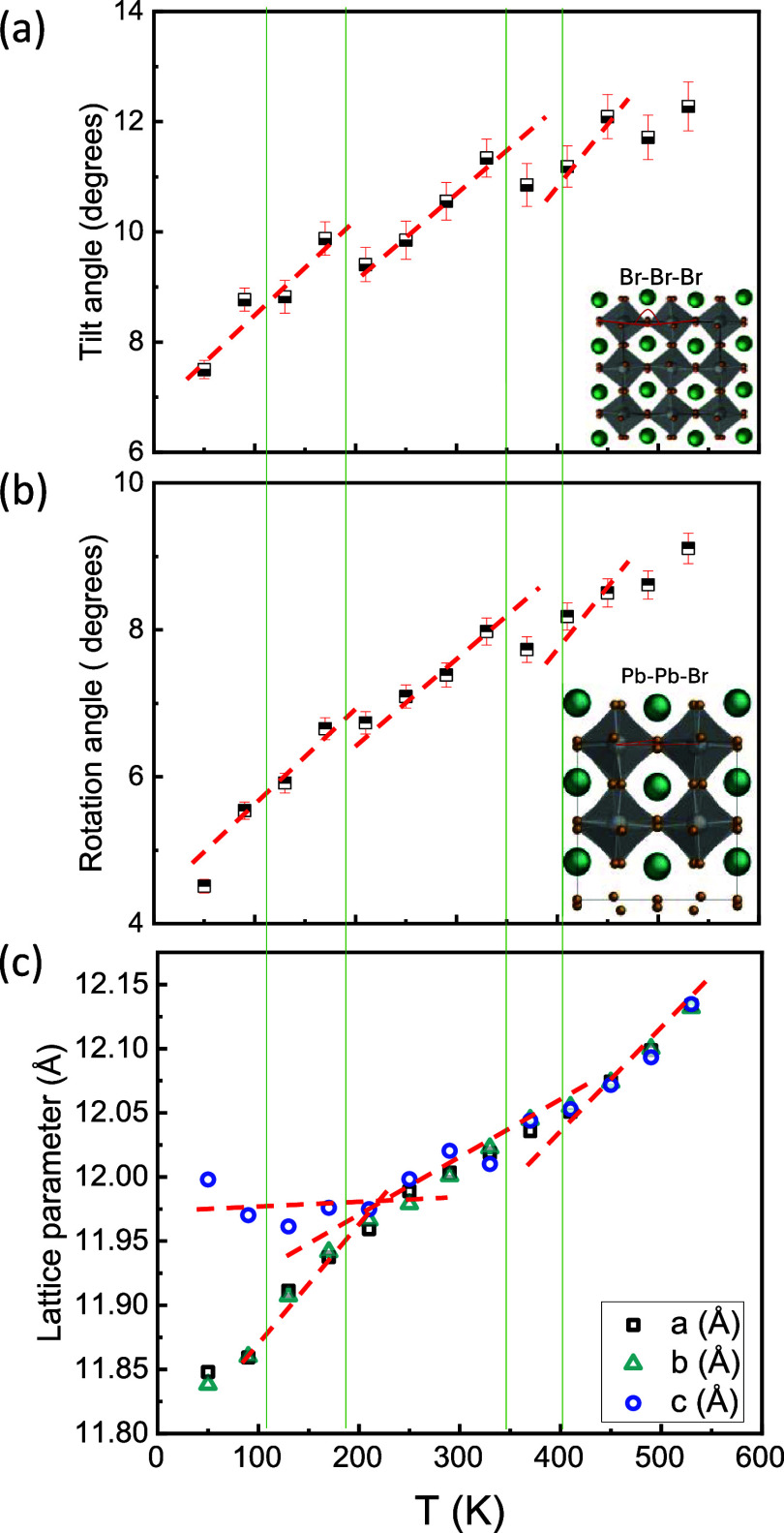
(a) Tilt angle, (b) rotation angle of the octahedra [PbBr_6_], and (c) lattice parameters for CsPbBr_3_ as a function of temperature calculated from MD simulations. The red lines in panels (a)–(c) along with the vertical green lines serve as an eye guide only. The onset points marked by red dashed lines indicate phase transitions observed across temperature ranges. The insets in panels (a) and (b) show the methods to determine the tilt and rotation angles adapted with permission from refs (48) and (49), respectively.

The tilt and rotation angles of the octahedra [PbBr_6_] in CsPbBr_3_ were calculated from the coordinates of the atoms obtained from the trajectories at various temperatures using a Python code and are shown in Figure 6a,b. The tilt angle of the octahedra is 7.5° at 50 K. As temperature increases to 520 K, the value of the tilt angle shows a nonmonotonic behavior. It progressively rises from 50 to 90 K, followed by a decline between 90 and 130 K, and then a subsequent increase from 130 to 170 K. Another decrease in the tilt angle occurs at 210 K. From 210 to 330 K, the tilt angle steadily increases, but drops again at 370 K, before increasing once more to 410 K. Similar fluctuations are observed in the rotation angle of the octahedra [PbBr_6_]. These abrupt changes in the tilt and rotation angles correspond to shifts in atomic movements and align with structural transitions discussed later. A careful analysis of the tilt and rotation angles shows nonzero values at 410 K and temperatures above that. The tilt and rotation angles should be zero in an ideal cubic structure. A nonzero value of the tilt and rotation angles in a cubic structure corresponds to the dynamic tilting behavior of PbBr_6_ octahedra in CsPbBr_3_. The presence of the dynamic tilting in the cubic phase is also reported in other metal halide perovskites such as CsPbI_3_ and CsSnI_3_.^53,54^

Figure 6c illustrates the lattice parameters extracted from MD simulations derived from the trajectories of Cs, Pb, and Br atoms at various temperatures. At 410 K and higher temperatures, the three lattice parameters *a*, *b*, and *c* are equal, measuring 12.052 Å at 410 K, slightly larger than those calculated from XRD and reported in ref (31). As the temperature decreases from 410 K, these parameters gradually decrease and exhibit slight deviations from each other until 130 K, indicating a phase transition from cubic to monoclinic. As the temperature is reduced further, the values for *b* and *c* lattice parameters keep decreasing, but the lattice parameter *c* increases from 130 to 50 K, as shown in Figure 6c. While the absolute values of the calculated cell parameters are higher than experimental observations,^24,30^ these discontinued changes of cell parameters show the phase transitions, which are qualitatively consistent with the experimental observations.^24,25,29−31^

#### Local Structure as a Function of Temperature

**Figure 7:**
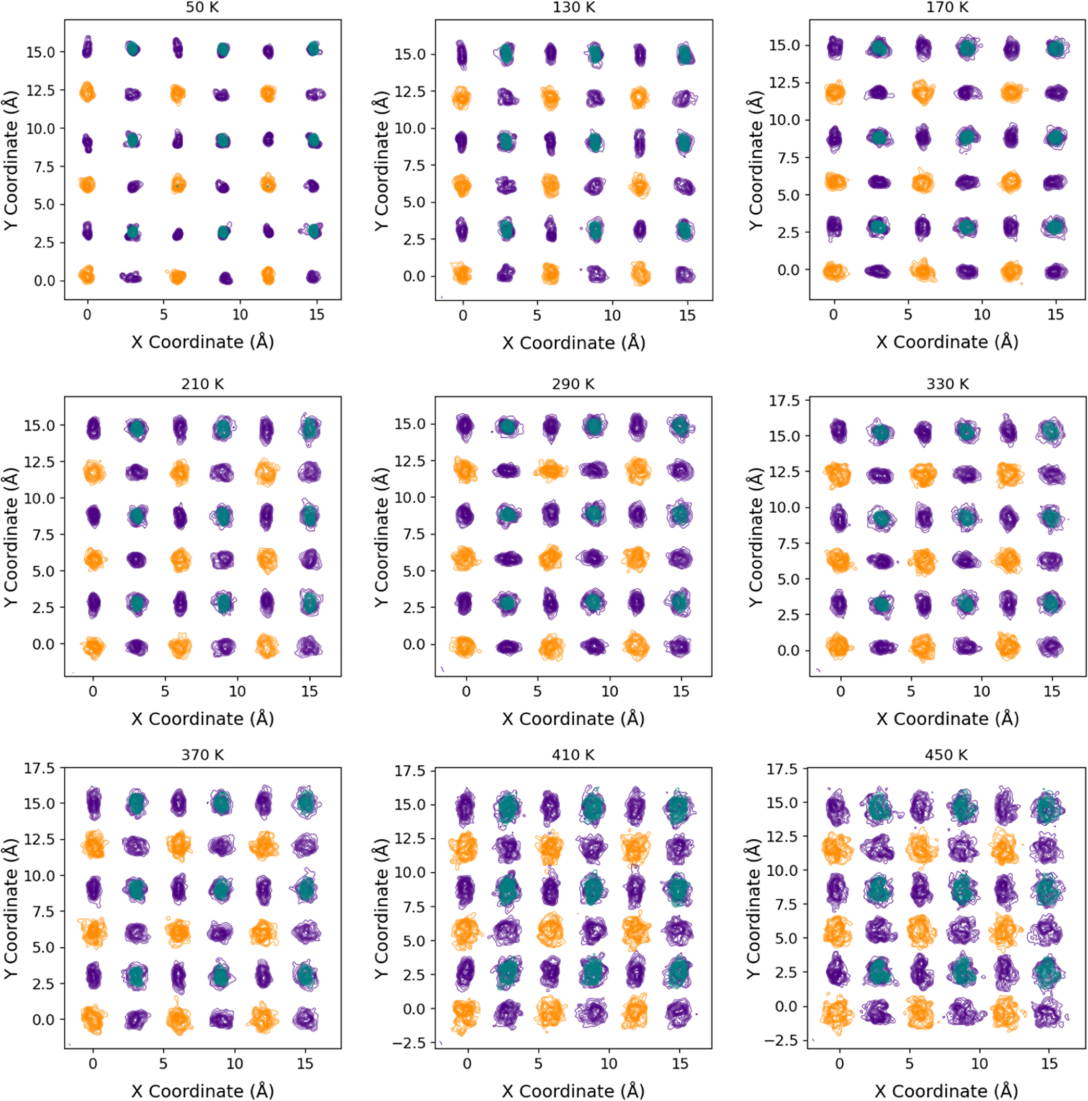
Atomic density contour maps for CsPbBr_3_ obtained from MD simulations at various temperatures. These maps represent Cs atoms in orange, Br atoms in violet, and Pb atoms in teal.

AIMD simulations reveal the changes in cell parameters and the local structure as temperature increases from 50 to 520 K. The atomic density contour maps at different temperatures are plotted in Figure 7. These maps were generated based on the atomic positions extracted from the trajectories obtained in MD simulations, using a Python code developed specifically for this project. Additionally, the trajectories were visualized dynamically using OVITO software^55^ to observe atomic movements. The atomic displacement is relatively small at low temperatures (e.g., 50 K). The atoms are confined to vibrate in small volumes. When examining the distribution of Br atoms, they appear to be more dispersed in space along neighboring Cs–Cs atoms than along neighboring Pb–Pb atoms. This indicates that the Br atoms primarily oscillate along the direction perpendicular to the line connecting the Pb–Pb atoms. This motion of the Br atoms is closely associated with the rotation of the octahedra [PbBr_6_] in CsPbBr_3_. This distribution pattern of Br atoms can be seen across all maps for all temperatures, with an increase in the distribution width with the rise of temperature. This is the signature for the presence of the rotation of the octahedra [PbBr_6_] from 50 to 530 K. Another interesting feature is the shape of the distribution of the atomic density of the Cs atoms at various temperatures. The shape of the Cs atom distribution is not isotropic at low temperatures (e.g., 50 K). The observed anisotropic shape of the Cs atom distributions at lower temperatures can be attributed to the inherent lower symmetry of the monoclinic crystal structure of CsPbBr_3_. In this phase, Cs atoms experience directional force fields that lead to preferential displacements along specific crystallographic axes. As temperature increases, the thermal energy enables atoms to overcome these directional constraints to some extent, causing a decrease in the observed anisotropic shape. In addition, there is a near-zero Cs atomic density at the center of the Cs site at most of the temperatures investigated. The shape of the distribution keeps changing with temperatures, leaving single or multiple gaps in the middle of the distribution; also, the distribution for Cs increases as the temperature rises to higher values. This result indicates that Cs atoms are rattling at the site and are too small concerning the space provided by the Pb–Br_6_ octahedra framework. The careful analysis of the atomic movements using the visualization software shows the presence of the Cs atom displacements from the center of the site. Another feature worth noting is the distribution of the atomic density of the Pb atoms as a function of temperature. The distribution of Pb atoms looks spherical from 50 to 330 K but slightly oval at 370 K and higher temperatures. A transition occurs between 330 and 370 K in the crystal structure, coinciding with the phase transition to cubic.

## Conclusions

Inorganic perovskite CsPbBr_3_ is a promising material for many applications, including radiation sensors. Understanding the crystal structure at the molecular scale is essential for developing advanced devices. The band gap calculated for CsPbBr_3_ at room temperature is 1.93 eV. Experimental crystal structure characterization is combined with ab initio molecular dynamics simulations to reveal the local structure, the octahedron’s rotation, and tilting as the crystal undergoes phase transitions. The simulations align with both our experimental findings and those documented in the existing literature, effectively replicating the principal characteristics of the phase transitions. It is confirmed that the crystal structure is transformed from one monoclinic to another monoclinic and then to cubic before melting. The stable phase crystal structure of CsPbBr_3_ at room temperature is monoclinic. Atomic density contour maps show the out-of-phase tilt and rotation of the octahedra in CsPbBr_3_. The tilt and rotation angles grow with temperature, with abrupt shifts occurring at transition temperatures. The current findings demonstrate that ab initio molecular dynamics can accurately replicate phase transitions, offering valuable insights into the electronic and molecular structures of the material. This comprehension is crucial for advancing device development across multiple applications.

## Supporting Information Available

Weak diffraction spots for SCXRD at 300 K; DOS for Cs, Pb, and Br; a different side view for the isosurface of the electron charge density, electron localization function, top of the valence band, and bottom of the conduction band; crystallographic data at 300 K; and structural parameters from SCXRD and ADP calculated from simulations (PDF)
