# Supplementary material for: Mapping and Characterization of Local Structures of CsPbBr3
Source: ACS Omega. 2024 Aug 9;9(33):35789–97. doi: 10.1021/acsomega.4c04354 (PMC11339804; doi:10.1021/acsomega.4c04354)
Supplement: Supplementary file 1 — ao4c04354_si_001.pdf [file ao4c04354_si_001.pdf]

Supporting Information

Mapping and characterization of local structures of $CsPbBr_3$

Tahira Khan[†, *], Sviatoslav Baranets[‡], Manas R. Gartia[§], Jianwei Wang[⊥, #, *] and Jyotsna Sharma[†]

[†] Department of Petroleum Engineering, Louisiana State University, Baton Rouge, Louisiana 70803, United States
[‡] Department of Chemistry, Louisiana State University, Baton Rouge, Louisiana, 70803, United States
[§] Department of Mechanical and Industrial Engineering, Louisiana State University, Baton Rouge, Louisiana 70803, United States
[⊥] Department of Geology and Geophysics, Louisiana State University, Baton Rouge, Louisiana 70803, United States
[#] Center for Computation and Technology, Louisiana State University, Baton Rouge, Louisiana 70803, United States

*Corresponding author; email: jianwei@lsu.edu, tkhan@lsu.edu

List of Figures

Figure S1 Weak diffraction spots with half-integer values observed for the precession image generated for the orthorhombic cell at 300 K. Page# S3

Figure S2 DOS for Cs (a), Pb (b), and Br (c) calculated from DFT. Page# S4

Figure S3 A different side view of (a) the calculated charge density (isosurface value of 0.03 e$Å^{-3}$) and (b) the Electron localization function (isosurface value of 0.85 e$Å^{-3}$) for $CsPbBr_3$. The red color corresponds to the crosscuts at the atoms in (a) and (b) while the yellow/green color stands for the electron accumulation. (c) Charge distribution at the valence band calculated over -3.5 eV to Fermi level and (d) at the conduction band calculated over 2 eV to 7 eV while the isosurface values of 0.10 e$Å^{-3}$ and 0.01 e$Å^{-3}$, respectively are used. Page# S5

Figure S4. Atomic Displacement parameters (ADP) are calculated by MD simulations as a function of temperature. Page# S9

List of Tables

Table S1. Selected crystallographic data and structure refinement details for monoclinic and orthorhombic refinement of $CsPbBr_3$. Ag Kα, λ = 0.56086 Å) Page# S6

Table S2. Temperature-dependent unit cell parameters for the CsPbBr3 obtained from SCXRD data. Page# S7

Table S3. Fractional atomic coordinates and equivalent anisotropic displacement parameters *Ueq* for $CsPbBr_3$ (300K data collection). Page# S8

Table S4. Atomic Displacement parameters (ADP) are calculated by MD simulations as a function of temperature. Page# S9

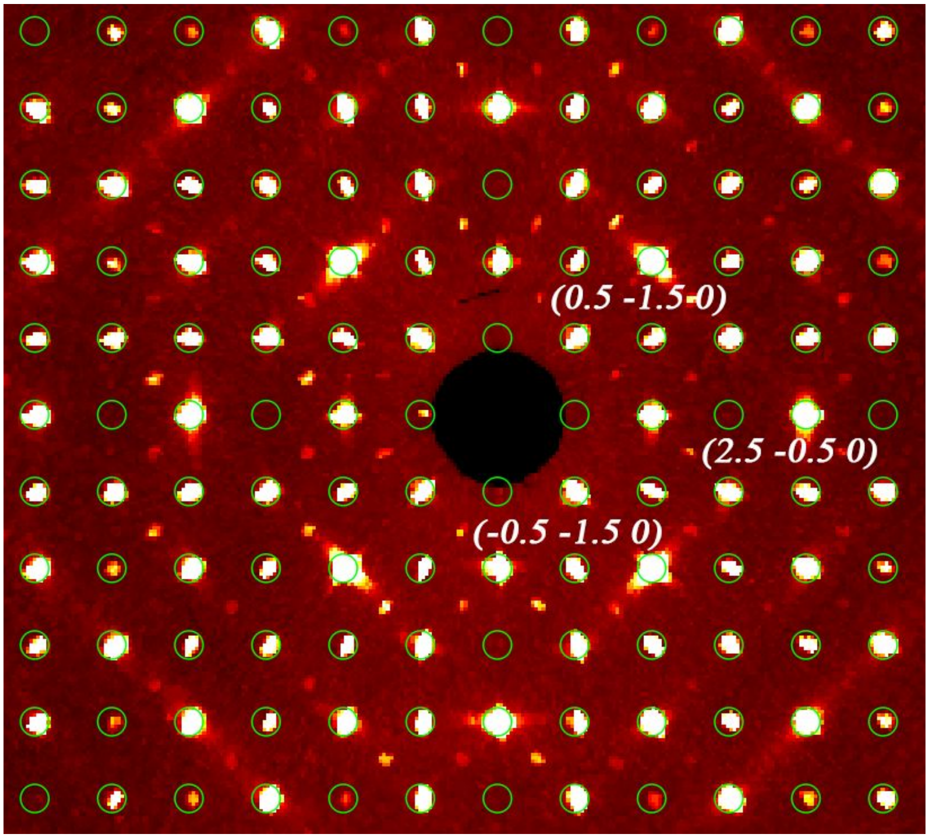

Figure S1 Weak diffraction spots with half-integer values observed for the precession image generated for the orthorhombic cell at 300 K.

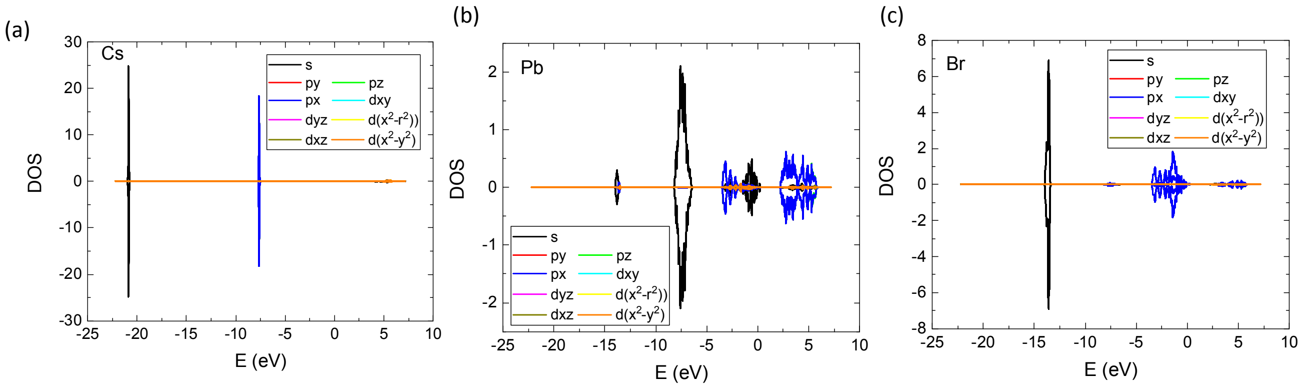

Figure S2 DOS for Cs (a), Pb (b), and Br (c)calculated from DFT.

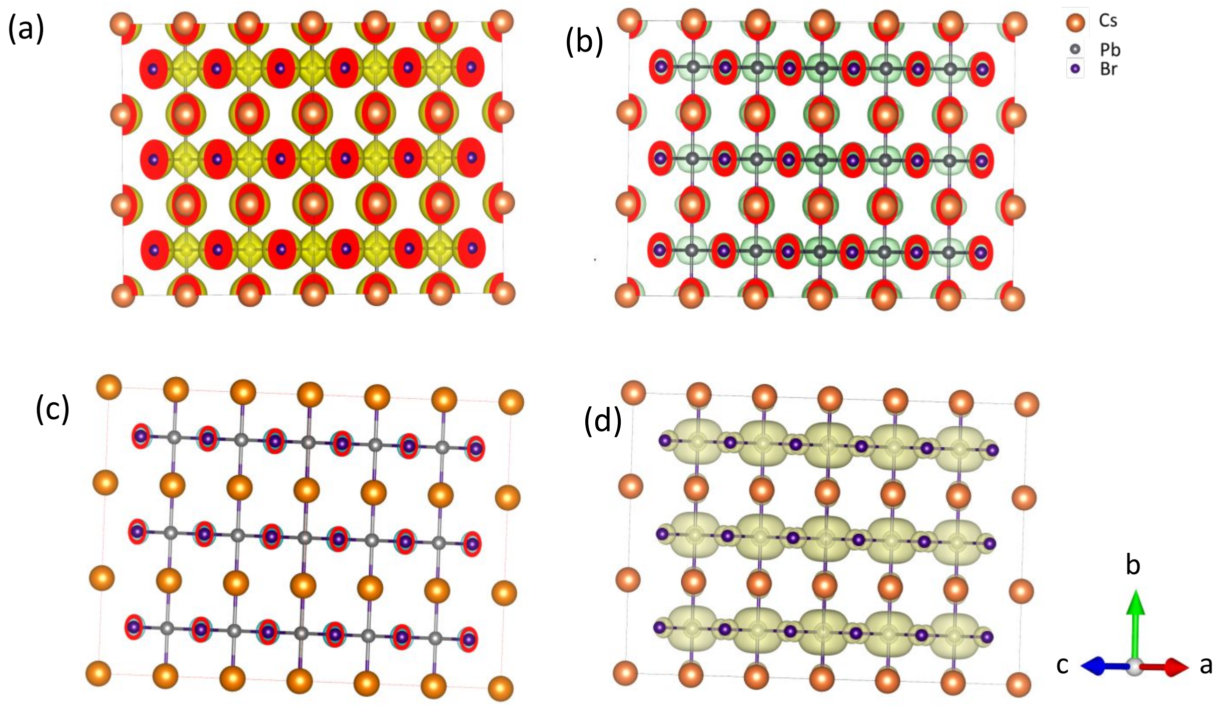

Figure S3 A different side view of (a) the calculated charge density (isosurface value of 0.03 eÅ^{-3}) and (b) the Electron localization function (isosurface value of 0.85 eÅ^{-3}) for $CsPbBr_3$. The red color corresponds to the crosscuts at the atoms in (a) and (b) while the yellow/green color stands for the electron accumulation. (c) Charge distribution at the valence band calculated over -3.5 eV to Fermi level and (d) at the conduction band calculated over 2 eV to 7 eV while the isosurface values of 0.10 eÅ^{-3} and 0.01 eÅ^{-3}, respectively are used.

Table S1. Selected crystallographic data and structure refinement details for monoclinic and orthorhombic refinement of $CsPbBr_3$. Ag Kα, λ = 0.56086 Å)

**Space group**	***Pmna***[b]	***Pm***	***Pm***
**Chemical formula**	$CsPbBr_3$		
**fw/g mol^{-1}**	579.83		
***a*/(Å)**	8.2166(2)	11.6455(3)	11.6366(4)
***b*/(Å)**	11.7458(3)	11.7466(3)	11.7392(4)
***c*/(Å)**	8.2494(2)	11.6424(3)	11.6353(4)
***β*/°**	90	90.230(1)	90.343(1)
***V* (Å^3)**	796.15(3)	1592.61(7)	1589.40(9)
Z	4	8	8
***T*, K**	300(2)		280(2)
**$\rho_{cal.}$/g cm^{-3}**	4.84		4.85
**μ(Ag K_α)/ cm^{-1}**	218.22		218.66
**Collected/independent reflections**	32174/1304	65308/10026	55876/7946
**R_1 ($I>2\sigma_{(I)}$)[a]**	0.032	0.042	0.052
**wR_2 ($I>2\sigma_{(I)}$)[a]**	0.082	0.084	0.103
**R_1 (all data)[a]**	0.036	0.082	0.093
**wR_2 (all data)[a]**	0.085	0.102	0.123
**$\Delta\rho_{max,min}$/e$^-$·Å^{-3}**	2.0/–3.7	3.1/–2.3	3.1/–3.2
**CCDC Code**	–	2350883	2350884

[a] $R_1 = \Sigma \mid |F_o|-|F_c| \mid / \Sigma|F_o|$. $wR_2 = \{\Sigma [w(F_o^2 - F_c^2)^2] / \Sigma wF_o^4\}^{1/2}$, $w = 1/[\sigma^2(F_o^2) + (AP)^2 + (BP)]$, where $P = (F_o^2 + 2F_c^2)/3$; A and B are weight coefficients.

[b] Orthorhombic unit cell was yielded by excluding weak half-integer reflections from the 300 K data collection.

CCDC deposition numbers 2350883 and 2350884 contain full supplementary crystallographic data for $CsPbBr_3$ collected at 300(2) K and 280(2) K, respectively. CIF files can be obtained free of charge via https://www.ccdc.cam.ac.uk/structures/, or by emailing data_request@ccdc.cam.ac.uk, by contacting The Cambridge Crystallographic Data Centre – 12 Union Road, Cambridge CB2 1EZ, U.K., fax +44 1223 336033.

Table S2. Temperature-dependent unit cell parameters for the $CsPbBr_3$ obtained from SCXRD data

**Temperature**	**350 K**	**300 K**	**280 K**	**250 K**	**200 K**	**100 K**
***a*, Å**	11.6833(15)	11.6455(3)	11.6366(4)	11.6343(11)	11.6175(13)	11.5570(14)
***b*, Å**	11.7597(12)	11.7466(3)	11.7392(4)	11.7248(8)	11.7098(10)	11.6663(12)
***c*, Å**	11.6718(13)	11.6424(3)	11.6353(4)	11.6090(9)	11.5910(11)	11.5476(13)
***β*, °**	90.027(4)	90.230(1)	90.343(1)	90.539(3)	91.087(4)	92.285(4)

Table S3. Fractional atomic coordinates and equivalent anisotropic displacement parameters U_{eq} for $CsPbBr_3$ (300K data collection).

Atoms	Site	*x*	*y*	*z*	U_{eq}[a] (Å^2)
**Cs1**	1*b*	0.2841(5)	1/2	0.7575(7)	0.068(1)
**Cs2**	1*b*	0.2913(5)	1/2	0.2627(6)	0.070(1)
**Cs3**	1*b*	0.7873(5)	1/2	0.2571(6)	0.067(1)
**Cs4**	1*b*	0.7915(5)	1/2	0.7705(6)	0.067(1)
**Cs5**	1*b*	0.2544(8)	0	0.2911(6)	0.095(2)
**Cs6**	1*b*	0.2630(6)	0	0.7811(8)	0.096(3)
**Cs7**	1*b*	0.7551(7)	0	0.7883(6)	0.084(2)
**Cs8**	1*b*	0.7633(6)	0	0.3030(8)	0.088(2)
**Pb1**	2*c*	0.0200(1)	0.2499(1)	0.0283(1)	0.025(1)
**Pb2**	2*c*	0.0200(1)	0.2499(1)	0.5283(1)	0.024(1)
**Pb3**	2*c*	0.5199(1)	0.2499(1)	0.5284(1)	0.024(1)
**Pb4**	2*c*	0.5201(1)	0.2499(1)	0.0283(1)	0.024(1)
**Br1**	2*c*	0.0644(6)	0.2726(4)	0.7794(5)	0.068(1)
**Br2**	2*c*	0.2705(4)	0.2752(3)	0.4873(5)	0.060(1)
**Br3**	2*c*	0.2710(5)	0.2182(4)	0.0703(7)	0.080(1)
**Br4**	2*c*	0.4781(5)	0.2241(4)	0.7798(5)	0.066(1)
**Br5**	2*c*	0.5633(6)	0.2763(4)	0.2806(5)	0.068(1)
**Br6**	2*c*	0.7706(4)	0.2736(3)	0.9856(5)	0.054(1)
**Br7**	2*c*	0.7711(5)	0.2553(4)	0.5707(7)	0.082(1)
**Br8**	2*c*	0.9783(5)	0.2275(4)	0.2790(5)	0.068(1)
**Br9**	1*b*	−0.0033(7)	1/2	0.5188(10)	0.079(2)
**Br10**	1*b*	0.0461(8)	1/2	0.0556(9)	0.078(3)
**Br11**	1*b*	0.4947(7)	1/2	0.0104(8)	0.062(1)
**Br12**	1*b*	0.5402(8)	1/2	0.5620(9)	0.076(2)
**Br13**	1*b*	−0.0026(8)	0	0.0133(12)	0.092(3)
**Br14**	1*b*	0.0346(11)	0	0.5550(8)	0.105(4)
**Br15**	1*b*	0.5022(8)	0	0.5068(9)	0.073(2)
**Br16**	1*b*	0.5535(9)	0	0.0578(8)	0.075(2)

[a] *Ueq* is defined as 1/3 of the trace of the orthogonalized *Uij* tensor.

Table S4. Atomic Displacement parameters (ADP) are calculated by MD simulations as a function of temperature.

T (K)	ADP for Cs (Å2)	ADP for Pb (Å2)	ADP for Br (Å2)
**50**	0.03705	0.01	0.01896
**90**	0.03851	0.01373	0.03204
**130**	0.06628	0.03347	0.06286
**170**	0.07279	0.0316	0.06912
**210**	0.09298	0.05202	0.09328
**250**	0.1168	0.06281	0.11242
**290**	0.10847	0.0518	0.10156
**330**	0.12475	0.06182	0.11774
**370**	0.13341	0.07035	0.12882
**410**	0.18053	0.11076	0.16881
**450**	0.19786	0.12643	0.19253
**490**	0.36595	0.2873	0.35696
**530**	0.23461	0.13763	0.21836

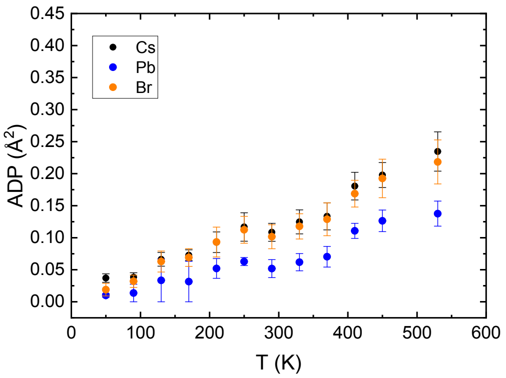

Figure S4. Atomic Displacement parameters (ADP) are calculated by MD simulations as a function of temperature.
